# Reconstruction of an Entire Phalanx

**Published:** 1850-05

**Authors:** Frank H. Hamilton

**Affiliations:** One of the attending Surgeons of the Buffalo Hospital of the Sisters of Charity


ART. III.—*Reconstruction of an Entire Phalanx.* Reported by FRANK H. HAMILTON, one of the attending Surgeons of the Buffalo Hospital of the Sisters of Charity.

Catharine Dolen, aged 24; admitted Dec. 25, 1849. Thumb.—The bone was necrosed, and on the 29th I extracted the phalanx entire. The inflammation having considerably subsided by the third of January, five days after the operation, I applied a tape roller the whole length of the thumb, and moderately tight. This was continued with occasional intermissions during two months, when a new phalanx was found to have been formed, of the same length and breadth, and form, as the original phalanx; the articulating surface was also reformed, and the flexor and exterior tendons so attached as that the motions of the joint were perfect.

This result is not now for the first time discovered. More than a year since, Prof. Dudley, of Lexington, informed me in a private communication, that he had been able to reconstruct a phalanx, where the bone had been entirely removed, by the continued application of the roller. To Dr. Dudley, therefore, is the profession indebted for this interesting pathological discovery.

We have many times seen the bone reconstructed, where a *portion* only of the whole was removed; as when the bone was partially destroyed by necrosis or caries and exfoliated, the deficiency has soon been supplied, so that the form, size, and functions were again restored. Especially has this happened when the process of necrosis was slow. Nor ought we to have been surprised to find the entire phalanx reformed after a slow destruction of the original, since the new phalanx might have been commenced be-

*See Buffalo Med. Jour., October, 1848, p. 367.

fore the complete death of the old, and thus serve as a nucleus for a reformation. This may have happened often, and may have been noticed, but it is the complete reconstruction of a bone when it has been removed in its tololity by extraction, that excites our surprise, and which we have marked as a novelty, for I am not informed that after Dr. Dudley, any one except myself has demonstrated its practicability.

