# Errata

**Published:** 1850-05

**Authors:** 


*Errata.*—In the article communicated by Prof. Hamilton, page 980, the following errors occurred, which the reader is requested to correct:—

Line *first*, should have read "Catherine Dolen, aged 24, admitted Dec. 25, 1849," *with onychia osteosa of the last phalanx of the thumb.*

In the *second* line, for "extracted," read *expected;* in the last line of the same paragraph, for "exterior," read *extensor;* and in the third line from the end of the article, for "totolity," read *totality.*

